# Questioning Established Protocols for New and More Sustainable Syntheses of Nanomaterials

**DOI:** 10.1021/acs.langmuir.5c05054

**Published:** 2025-12-12

**Authors:** Jonathan Quinson

**Affiliations:** CICA-Centro Interdisciplinar de Química e Bioloxía, Facultade de Ciencias, 16737 Universidade da Coruña, Campus de Elviña, 15008 A Coruña, Spain

## Abstract

Advances in nanotechnologies rely on bulletproof synthetic protocols of nanomaterials. Bringing breakthroughs from fundamental nanoscience to real-life solutions requires cost-efficient and scalable strategies. Sustainability is not only driving modern societies but also increasingly shaping modern research, opening a range of opportunities as well as calling for new mindsets. This perspective suggests how to navigate and address in a timely manner all of these challenges at the same time by questioning established synthetic protocols of nanomaterials. In particular, the development of so-called surfactant-free colloidal syntheses of metal nanoparticles is a promising area of research, opening seldom explored avenues to comply with the principle of g*reen* and s*ustainable* chemistry, to develop a new understanding of nanoparticle formation, and ultimately lead to improved nanomaterials and technologies.

## Graphical abstract

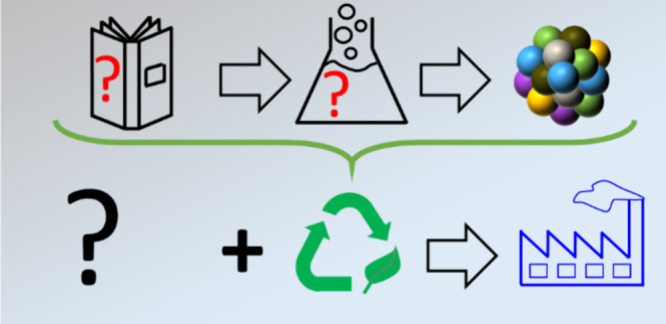

## Introduction

### Nanotechnology

Nanotechnologies are reshaping our societies. From electronics,^1^ medicine,^2^ sensing,^3^ chemical production,^4^ energy conversion^5,6^ all the way to water/air treatment,^7^ and many other areas of application,^8,9^ nanomaterials (NMs) are in increasing demand. Nevertheless, relatively few of the many NMs explored and reported to date found their way to the larger-scale production to become part of impactful technologies.^10–12^ In parallel, sustainability considerations are increasingly shaping modern society and modern research.^13^ So, how can one address so many challenges at the same time? The answer might come from considering that addressing one challenge is not exclusive to addressing another. Indeed, developing sustainable syntheses or NMs, involving fewer, safer, less energy, and less waste-generating steps, is likely to lead to more affordable, scalable, and relevant processes, as well as provide new fundamental insights.

### Sustainability

*Green* and *Sustainable* chemistry are certainly problematic that are no not foreign to NM synthesis. A range of reviews covers in length the topic^14–16^ to develop safer and more efficient *green* processes and/or use *sustainable* approaches, for instance, preferring renewable resources and low-energy strategies. At the same time, there is a general agreement that NM synthesis (being *green* or not) remains a ‘black-art’, heavily based on trials-and-errors where many ‘tricks’ are involved to get the expected outcomes.^4,12^ This is due to the inherent complexity of the field. Despite numerous synthetic protocols reported for various NMs,^14,17,18^ and despite an increasing understanding of the formation mechanism(s) of most NMs (which is often considered the key toward fully controlled syntheses),^19,20^ there is still a lot to learn.^17,21,22^ Although it might seem counterintuitive and *limiting* to develop *greener* and more *sustainable* syntheses of NMs—in the sense that restricting oneself to some solvents, reagents, temperatures, etc.,^23,24^ might bring more limitations than opportunities—the outcomes might actually be a deeper understanding and control over NM formation and properties.

### A Slow Process

Most syntheses of NMs have been refined over the years. It is indeed challenging to grasp from the first reports *what matters*. A textbook example is maybe the worldwide implemented synthesis of gold (Au) nanoparticles (NPs) by the Borowskaja–Turkevich–Frens method, reported in 1934 by Borowskaja,^25,26^ revisited in 1951 by Turkevich,^27^ by Frens in 1973,^28^ and popularized over the years due to its simplicity since it only requires water, sodium citrate, and a gold precursor.^17,29^ The method has been improved from a direct method (adding citrate last) to a reverse method (adding the gold precursor last) in 2011,^30,31^ showing the importance of the order of the addition of the chemicals, and has been constantly reinvestigated since from different angles, giving room for debate on the (many) roles of the otherwise few chemicals needed.^17,32^ The Borowskaja–Turkevich–Frens synthesis became popular due to its simplicity to execute, but at the same time, its complexity to understand. This synthesis is therefore a good example of how *easier* (practical) is not always *simpler* (fundamental understanding).

### Challenges

It can therefore be argued that a range of syntheses, already reported or to be developed, might be overlooked model systems for future breakthroughs, both in our understanding of NM formation and synthesis, and also to unlock the keys to real-life applications. An underlying challenge is how to avoid spending ca. 80 years, as for the Borowskaja–Turkevich–Frens synthesis, to develop an “optimized” synthesis. This perspective first aims to highlight some challenges in reporting and developing syntheses of NMs. Second, this perspective shows how aiming for *greener* syntheses, by developing entirely new protocols or revisiting old protocols, can actually lead to a new understanding of NM syntheses. This perspective stresses how more chemically efficient methods can be directly relevant for fundamental and applied research. As an illustration, a focus is given to the example of so-called *surfactant-free* colloidal syntheses of metal NPs due to their wide range of applications. Last, future possible areas of development and new doors opening from the development of such methods are highlighted and discussed.

Interested readers will find elsewhere more detailed reviews on *Green* and *Sustainable Chemistry* for NMs.^14–16^ In particular, the goal here is not to cover all emerging or established strategies for NM syntheses. Dedicated work on alternatives to develop NMs can be found elsewhere, e.g., using enzymes or genetically engineered organisms,^33^ preferring widely available (bio)­materials,^34^ or using alternative solvents such as deep eutectic solvents^35^ or supercritical fluids.^36^ The focus here is more to highlight the need and benefits of curiosity and a mindset prone to question the *status quo* reached to date for most syntheses of NMs.

## Perspective

### Easier but Not Always Simpler: What *Does* Matter?

The synthesis of NMs can require an all range of chemicals and can be performed in an all range of ways. A common challenge in revisiting and/or developing a synthetic method for NMs is to identify *what matters*. It is often challenging on first reports to identify what truly has an influence on the outcome of a synthesis.^37^ It is often *a posteriori* that important experimental parameters are identified,^17^ often due to an increasing experience with the process gained over time. While the use of supporting information should help to best address this challenge, lengthy detailed protocols are far from being the norm. For instance, it is not often reported what final concentrations of chemicals were used. It is more common to state the amounts of chemicals selected, leaving it to the reader to recalculate and/or reinterpret all concentrations, for instance, and/or all the ratios between the different chemicals. In the same way, how the samples or stock solutions were stored, what containers were used, etc., would need to be more explicitly detailed.^38^ Until proven otherwise and systematically specified, all those parameters may (or may not) have a critical and complex influence, as schematized in Figure 1.

**Figure 1:**
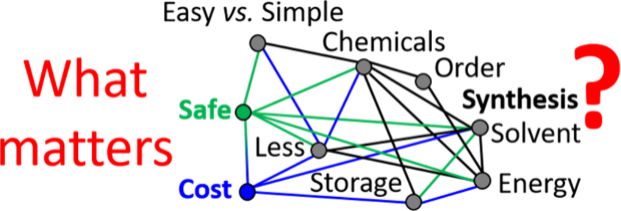
Developing new NMs and their synthesis: What matters? Designing an optimal synthesis of NMs requires finding a fine balance between safety, costs, waste generated, and/or sustainability considerations to obtain yet the right materials with the right properties.

One way to bypass this potential lack of information, and speed up the process of obtaining reproducible results widely implementable, would be to contact corresponding authors. Although there is no specific data on the response rate of corresponding authors for the nanosciences, by extrapolation of what was evaluated in biomedical fields,^39^ there is roughly less than a 50% chance of getting an answer and obtaining more details on synthetic procedures. Fortunately, the awareness on the need for openly sharing good practices is increasing,^12^ but even more transparency could be desirable and certainly rewarding. Reporting more unambiguous, transparent, and intelligible protocols together with a wider openness on ‘tricks’ could be reported in the supporting information of publications. Being open to those details could also benefit from a different mindset from peers. Exposing upfront uncontrolled variables should not be regarded by the community (and especially by reviewers) as a lack of in-depth investigation (time, resource, and priority constraints are a reality of modern research) but as a mark of awareness aimed at facilitating the task for others to best reproduce and timely develop the work. By being as descriptive as possible, one provides the tools and the flags for others to pay attention to what could *possibly* matter. In this direction, video protocols are a promising way to alleviate the related challenges and uncertainties.^40^ Finally, with the increasing use of artificial intelligence tools,^41^ it will be increasingly easier to not only compile but also present the similarities and differences between different protocols. This could ultimately help identify *hidden* or *overlooked* parameters. Therefore, explicit reporting of those parameters is expected to be rewarding for the all community in the long run. These practices (or lack of) explain in part the challenges in reproducing and improving NM syntheses.

It is definitely challenging to identify *what matters*. First, due to the practicalities mentioned above that relate to *how* the synthesis is performed and reported. Second, irreproducibility can come from the *what*, i.e., the nature and grades of chemicals, that is known to play a key role in reproducibility, but challenging to control and investigate in most cases.^12,42–44^ A third and maybe overlooked layer is the *which*, i.e., when various options are available, which one to choose? Many chemicals are expected to be interchangeable. For instance, in many syntheses, alkaline conditions might be needed.^45^ Typically, NaOH or KOH is used.^46^ However, the potential effect of the cation, e.g., using also LiOH, is rarely considered. We observed a strong effect of the countercation in alcohol-mediated syntheses of platinum (Pt), iridium (Ir), or Au NPs.^22,46,47^ More recently, we assessed this effect in citrate (XCt) and borohydride (XBH_4_) mediated syntheses, where X = Li, Na, or K. Li-based chemicals were found to lead to more stable colloidal dispersions of Au NPs.^48^ The stabilization of metal surfaces by counter cations due to stronger noncovalent interactions decreasing in the order Li^+^ > Na^+^ > K^+^ is a well-established phenomenon, e.g., in electrocatalysis, where KOH electrolytes are preferred to minimize cation-metal interactions,^49^ but surprisingly scarcely considered for colloidal syntheses.

In other words, if the question is ‘*does this chemical matter*?’ or ‘*does it matter if I do this step this way or that way*?’, or ‘*does it matter if I use this chemicals rather than this one*?’ and if the answer is not in the literature, or is ‘*I do not think so’*, it should actually be considered that it *does* matter until being proven otherwise. This point is in particular addressed to the younger researchers, who often have the feeling that syntheses, recipes, and methods (especially those developed by the hosting group) are well-established and set in stone. It is rarely the case. It is only with clear answers to questions such as ‘*do I really need this chemical, and if so, in which amount?*’ that will we be able to efficiently advance the field of NM synthesis. Answering those questions starts with transparency in reporting what has been investigated but also in some (most?) cases, what has not or could not be investigated (yet).

### Sustainability for New Breakthroughs

The importance of considering, mapping, and eventually addressing the possible effects of seemingly irrelevant experimental parameters becomes even more important on *easier* syntheses, i.e., syntheses requiring few chemicals and few steps. An acute attention to detail should be given for those emerging syntheses because the effects of small changes in protocols are more likely to be very pronounced, as exemplified below. A good example is the importance of water purity. Water is an increasingly important resource to societies and in increasing demand for nanotechnologies as a *green* solvent.^23,50^ It became clear over the years that high-purity water is a must for a successful synthesis of various NMs, for example, Au NMs.^18,42,43^ However, the use of high-purity water comes with a price, literally. The cost of purifying water increases with its purity, and it can be challenging to develop scalable methods based on water as a solvent. In this respect, although the degree of purity of the water will play a role, (re)­investigating the effect(s) of using lower purity water can be valuable.

Indeed, it is often by tuning the nature and/or concentrations of various chemicals that the size, shape, or structure of NMs can be tuned.^17,51–53^ At a practical level, this means that different amounts of potentially hazardous chemicals need to be considered. In contrast, given the importance of water as *a green* solvent, focusing on water purity can be a convenient option to revisit in order to tune NM properties, i.e., by an approach that does not involve changing the concentrations and/or amounts of any of the other *required* chemicals.^54^ Laser ablation is a good example of a *green* approach easily performed in lower-purity water.^55–57^ However, the method becomes sensitive to several parameters (on the laser itself) and the chemicals used. For a wider use by nonexperts, more classical colloidal syntheses obtained by reduction of metal precursors in the liquid phase would need to be developed.^19,20^ In this direction, water purity is a convenient knob that can be leveraged to tune the Au NP size. This approach was successfully implemented in a room temperature ethanol-mediated synthesis without any other chemicals than ethanol, water, a base, and a gold precursor. It is observed that the Au NP size increases as the water purity decreases.^54^ We also observed that Au NP sizes increase with the purity and the conductivity of water for Au NPs obtained by an approach that does not require any other chemicals than water, a gold precursor, and NaBH_4_.^58^

Despite the potential high costs of pure water, *easier* syntheses complying with the principles of *Green* and *Sustainable* chemistry are also more likely to find their way to a larger scale. *Easier* syntheses might help address the challenges in climbing the ladder of technology readiness levels (TRLs),^59,60^ as schematized in Figure 2. This is simply because such syntheses are expected to be overall cheaper and safer to perform, so they are more cost-efficient to scale. In relation to the TRLs, a better integration of techno-economic analysis (TEA) and life cycle assessment (LCA),^15^ including energy consumption, waste management, environmental impact, recycling, etc., is a key part of the development of more sustainable technologies.^61^ It can remain challenging to perform such analyses by nonexperts, but it can be expected that *easier* and more sustainable syntheses of NMs will also facilitate bridging this gap between areas of expertise.

**Figure 2:**
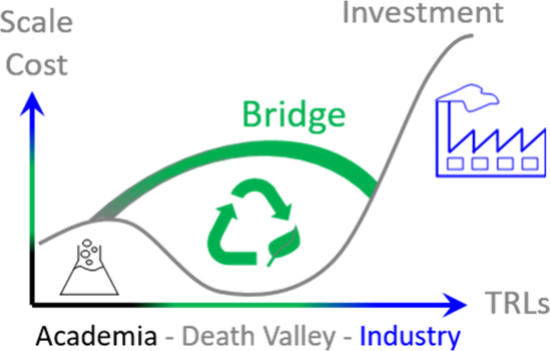
Sustainability as a bridge for knowledge transfer from academia and industry through various technology readiness levels (TRLs). While most fundamental findings struggle to find their way to a larger scale, sustainability can be a way forward to bridge the gap.

For instance, motivated by a will to not waste expensive Pt precursor, we could identify that an aged precursor could lead to the room temperature syntheses of Pt NPs in alkaline methanol or methanol–water mixtures, where heating up would be required with a fresh precursor.^62^ This incentive to minimize waste leads us to a potential strategy to reduce the overall cost related to inducing the synthesis. As another example, driven by the will to minimize volumes and yet obtained large amount of materials, we showed that surfactant-free syntheses of Au NPs can be performed a relatively high concentration of gold precursor HAuCl_4_ (0.5 mM) and yet the process scales up easily to 1 L.^47,63^ Surfactant-free syntheses of precious metal such as Pt obtained in alkaline monoalcohols were also successfully scaled to the kilogram scale due the simplicity of the approach requiring only a metal precursor, NaOH and methanol/ethanol.^64^

Another important aspect to consider to developing new sustainable colloidal syntheses of metal NPs is the reducing agent used.^24^ In a range of popular syntheses, strong and relatively harmful chemicals such as NaBH_4_ or hydrazine are used.^24,53^ Milder reducing agents include various chemicals with different chemical groups. In this direction, countless reviews cover so-called biogenic syntheses of NMs,^18^ and in particular for gold and silver NMs that easily form at room temperature due to the relatively high redox potential of the corresponding metal complex precursors.^47^ Almost any plant/fruit/food extract, fungi, microbes, or bacteria can act as a source of reducing agent and can account for NP formation and stabilization.^65^ However, such approaches suffer from several drawbacks. (i) The extraction of molecules requires organic solvents and/or generates waste. (ii) The species responsible for the reduction and/or stabilization can be challenging to identify, preventing further optimization. (iii) Biocompounds from different sources (e.g., different geographic origins) will have slightly different chemical compositions, which can influence the outcomes of a synthesis. (iv) The effect(s) of possible (and potentially many) chemical groups present in the bioderived extracts can influence biocompatibility and/or surface and/or catalytic properties in a way that is difficult to predict. This overall leads to the question of how scalable the production of NMs based on such resources could be.

To address this challenge, using well-defined simple molecules such as glycerol or ethylene glycol as a solvent and a source of reducing agent (typically under alkaline conditions) is an alternative. So-called polyols syntheses, extensively studied by Fievet at co-workers,^45^ have been shown to be relevant for a wide range of NM preparations.^66^ One drawback of polyols is the need to perform several steps to remove the viscous solvent before using the NPs. An emerging alternative is to substitute the polyols with monoalcohols such as methanol (despite its toxicity^23^) or ethanol, given that some reaction even proceeds using commercial spirits.^67^ An advantage of using such approaches with low boiling point solvents is to lead to simpler workup and a more direct use of the as-prepared NMs.^64,68^ At a larger scale, this would ultimately minimize processing costs and generate less waste.

A last point to consider in the direction to develop more sustainable syntheses is the recycling of the solvent and/or reducing agents. Indeed, in most cases the reducing agent is sacrificial^24^ and too few studies investigate the opportunities to recycle the solvent.^64,69^ Due to the relative scarcity of reports in this area, significant new insights can be expected in the future.

### Remove versus Substitute: When Less Is More

As stated and illustrated above, *Green* and *Sustainable* syntheses of NMs have been topics of interest for a while. However, in most cases, the focus is on substituting chemicals for less hazardous alternatives serving the same or similar purposes. In particular, for a range of protocols based on wet chemical methods, and in even more cases when performed in aqueous media, various additives are almost systematically claimed to be needed and are added for the sole purpose of stabilizing the NPs.^4,70^ Note that the Borowskaja–Turkevich–Frens synthesis is, in this sense, an elegant synthesis where the reducing agent also plays the role of stabilizer.

A more dramatic step beyond substitution would be to actually *remove* chemicals, and, in particular, those additives often derived from fossil fuels.^71^ Colloidal NP syntheses always need some sort of stabilization. However, the latter does not have to be achieved by chemicals added for that sole purpose.^55^ In this direction, surfactant-free synthesis is promising.^72^ A surfactant-free synthesis can be arguably defined as a synthesis where no other chemical than the metal precursor has a molar mass of 100 g mol^–1^. In other words, only small molecules are expected to play a role as both reducing agents and stabilizers.

Certainly, the use of *additives*/*surfactants*/*stabilizers*/*shape directing agents*/*capping agents*/*ligands* is a valuable tool to achieve complex structures, such as chiral NMs.^73,74^ It cannot be denied that such strategies led to several breakthroughs in material science and catalysis.^5,75^ However, the large-scale applicability of the methods might be questionable.^12^ First, due to the use of potentially harmful chemicals such as cetyltrimethylammonium bromide (CTAB), often used, for instance, to obtain Au nanorods.^12^ Second, a range of applications would benefit from clean surfaces, in catalysis and/or medicine, when ligand exchange is required.^72^ In other words, the benefits of developing advanced and complex materials with size and/or shape control, leading to different structures and thus to materials with tuned properties and activity but protected by such additives, might not always balance the benefits of developing a less-defined shape with cleaner and more active surfaces. In this respect, *less* is *more*. A striking example in the case of catalysis is the use of polyvinylpyrrolidone (PVP) passivating most surfaces,^47^ and yet it is one of the common additives used to stabilize NPs.^76^ The additives can be removed but typically via intensive chemical or energy-demanding steps, often poorly scalable, and with typically moderate success.^72^ A strategy to address this challenge will be to get rid of *unnecessary* chemicals altogether. The challenge remains to identify what chemicals are then *necessary*.

The polyol synthesis is a good example here. Wang et al. showed that actually no stabilizers are needed to produce a range of precious metal NPs using alkaline ethylene glycol.^77^ Conveniently, monoalcohols can be substituted for polyols,^64^ allowing a reaction at a lower temperature, in low-boiling-point solvents, more easily processed (e.g., evaporated) at a lower temperature to prepare supported NP readily active for catalysis.^78^ In the case of Au NPs, the synthesis even proceeds at room temperature in an alkaline mixture of water and ethanol,^47^ as illustrated in Figure 3. Conveniently, the reaction can be performed at relatively low amounts of base and ethanol, making the synthesis extremely easy, potentially even simpler than the Borowskaja–Turkevich–Frens method. As *easy* does not mean *simple*, the experimental window where the synthesis is successful is narrower with the monoalcohols than with the polyol, and a strong effect of light is to be expected.^54^ This last example illustrates the opportunities opened by simplifying reactions with the chance to lead to a better understanding of the influence of parameters (such a light) on the formation of NMs. Another example is the work of Astruc and co-workers who showed that in the aqueous NaBH_4_-mediated synthesis of gold NPs, there is no need for extra stabilizers.^53,58^ There are therefore already several examples of synthesis where an excess of chemicals is used, in the sense that the synthesis could proceed equally well by removing completely some species often reported to be *needed*, such as a range of *surfactants* or *stabilizers*.

**Figure 3:**
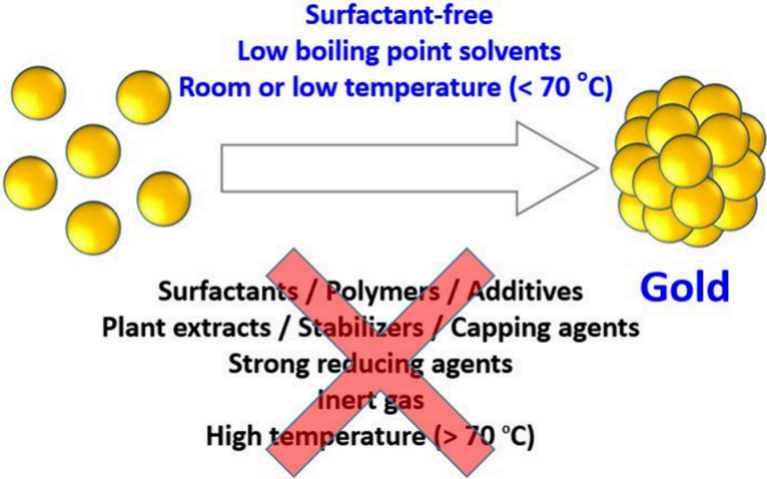
Example of synthetic strategies of surfactant-free colloidal gold NPs performed in alkaline mixtures of monoalcohols or polyols and water and its advantages compared to state-of-the-art approaches. As opposed to most reported recipes, this approach is simpler, safer, and thus generally easier to implement and scalable.

## Moving Forward

Beyond a call to revisit a range of syntheses and identify truly what is minimally needed to achieve a given outcome, surfactant-free syntheses of (metal) NPs offer a promising combination of properties. (i) They are additive-free, but additives can be added. In this sense, it provides a useful *blank* or *control* experiment, very much missing to date, to assess the role and need for chemicals selected empirically until now.^79^ (ii) Some of those syntheses can be performed at low or room temperature, which greatly facilitates a range of real-time monitoring.^37,80^ For instance, performing reactions at room temperature facilitates coupling UV–vis, pH measurements, and/or open circuit potentials. This could lead to new insights into NM formation.^80,81^ (iii) The preference for safer chemicals allows performing the synthesis in various laboratory environments, giving flexibility and modulability, e.g., at synchrotron facilities for advanced characterization.^82^ (iv) All together, the approach leads to a relatively high throughput,^83^ with the potential to be more easily enhanced by automation,^84^ thus tackling the main bottleneck of data-driven development of colloidal syntheses and implementing artificial intelligence and machine learning.^85,86^ (v) Ultimately, the syntheses performed in low viscosity solvents such as in aqueous media are ideally compatible with flow syntheses with a promising potential for scale up.^10,87^ For all those features, such model syntheses are, in addition, promising educational material.^88^

The combination of the previously unique properties is envisioned to contribute to a paradigm shift in nanotechnology. For instance, colloidal-surfactant-free syntheses are probably an untapped resource to advance material science. Such a synthesis is an ideal blank or control that was crucially missing to date to understand what chemicals are truly needed (see (i) above). Furthermore, it is anticipated that due to their ease of performance, the combination with various probes will allow real-time monitoring of various parameters. Real-time monitoring will ultimately provide information about the formation mechanism(s) of NMs. In addition, it will allow acting on the synthesis *as it proceeds*, in a dynamic way, as schematized in Figure 4. For instance, additives (e.g., for shape control) or precursors (e.g., for multimetallic NMs) could be added at selected and well-identified stages of the reaction. Such a concept will allow us to move away from the classical black box approach and establish *when* various chemicals are actually needed. This would lead to a better understanding of the role of various chemicals and ultimately to the development of protocols with an adjusted and *minimal* amount of chemicals for a desired outcome, assuming, until proven otherwise, that most recipes to date use an excess of chemicals. An excess of chemicals not only leads to more expensive protocols but also generates waste and/or requires energy-consuming post-treatments and washes. More controlled syntheses with fewer chemicals could then lead to the development of increasingly advanced materials by more efficient strategies, more directly relevant for large-scale preparation.

**Figure 4:**
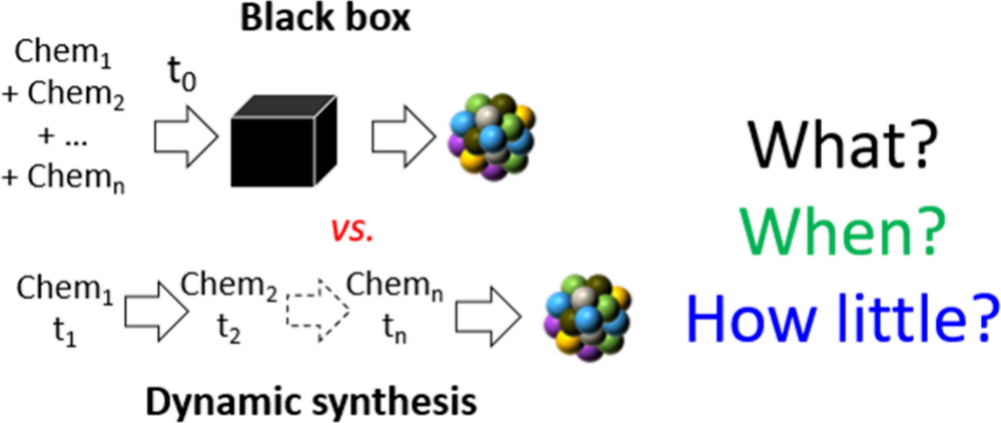
Opportunities for a paradigm shift in NM synthesis by developing more easy and more sustainable syntheses. By simplifying syntheses, opportunities open to understand better the roles of chemicals, therefore use the minimal amount of chemicals at the right time, for the right purpose, and therefore develop new synthesis concepts.

## Conclusions

Although the examples detailed above might not be applicable to all NMs, it is hoped that this perspective illustrates how questioning apparently *established* protocols can lead to significant inspiration for a new understanding of NM syntheses.

It is maybe too often reported and presented that a synthesis is “bulletproof.” Being more open to the possible uncontrolled variables (for the researchers) and more open to seeing those variables being reported or commented on (for reviewers and publishers) could help to more timely understand *what matters*, what is (minimally) *needed*, *when*, and *how*. This is especially increasingly relevant with the rise of open access databases and tools to navigate the related data to ultimately identify *hidden* key parameters.^89^ It is only with this knowledge at the end that we will be able to develop *optimal* syntheses, allowing us to produce in a reproducible way a given target, size structure, and composition by the most efficient and controlled routes.

It is hoped that the examples provided motivate researchers to rethink protocols toward more sustainable strategies, given the multiple potential positive outcomes not only for fundamental but also applied research toward more impactful nanotechnologies.

## References

[1] Kovalenko M. V., Manna L., Cabot A., Hens Z., Talapin D. V., Kagan C. R., Klimov V. I., Rogach A. L., Reiss P., Milliron D. J. (2015). Prospects of Nanoscience with Nanocrystals. ACS Nano.

[2] Rai M., Ingle A. P., Birla S., Yadav A., Dos Santos C. A. (2016). Strategic
role of selected noble metal nanoparticles in medicine. Crit. Rev. Microbiol..

[3] Tabatabaei M. S., Islam R., Ahmed M. (2021). Applications of gold
nanoparticles
in ELISA, PCR, and immuno-PCR assays: A review. Anal. Chim. Acta.

[4] Cargnello M. (2019). Colloidal
Nanocrystals as Building Blocks for Well-Defined Heterogeneous Catalysts. Chem. Mater..

[5] Guntern Y. T., Okatenko V., Pankhurst J., Varandili S. B., Iyengar P., Koolen C., Stoian D., Vavra J., Buonsanti R. (2021). Colloidal Nanocrystals as Electrocatalysts
with Tunable
Activity and Selectivity. ACS Catal..

[6] Seh Z. W., Kibsgaard J., Dickens C. F., Chorkendorff I. B., Norskov J. K., Jaramillo T. F. (2017). Combining
theory and experiment in
electrocatalysis: Insights into materials design. Science.

[7] Pradeep T., Anshup (2009). Noble metal nanoparticles
for water purification: A critical review. Thin
Solid Films.

[8] Paidari S., Ibrahim S. A. (2021). Potential application of gold nanoparticles
in food
packaging: a mini review. Gold Bull..

[9] Jones W., Gibb A., Goodier C., Bust P., Song M., Jin J. (2019). Nanomaterials in construction - what is being used, and where?. Proceedings of the Institution of Civil Engineers-Construction
Materials.

[10] Saldanha P. L., Lesnyak V., Manna L. (2017). Large scale syntheses of colloidal
nanomaterials. Nano Today.

[11] Charitidis C. A., Georgiou P., Koklioti M. A., Trompeta A.-F., Markakis V. (2014). Manufacturing
nanomaterials: from research to industry. Manufacturing
Review.

[12] Scarabelli L., Sanchez-Iglesias A., Perez-Juste J., Liz-Marzan L. M. (2015). A ″Tips
and Tricks″ Practical Guide to the Synthesis of Gold Nanorods. J. Phys. Chem. Lett..

[13] Freese T., Elzinga N., Heinemann M., Lerch M. M., Feringa B. L. (2024). The relevance
of sustainable laboratory practices. RSC Sustainability.

[14] Duan H. H., Wang D. S., Li Y. D. (2015). Green chemistry
for nanoparticle
synthesis. Chem. Soc. Rev..

[15] Hutchison J. E. (2016). The Road
to Sustainable Nanotechnology: Challenges, Progress and Opportunities. ACS Sustainable Chem. Eng..

[16] Gilbertson L. M., Zimmerman J. B., Plata D. L., Hutchison J. E., Anastas P. T. (2015). Designing nanomaterials
to maximize performance and
minimize undesirable implications guided by the Principles of Green
Chemistry. Chem. Soc. Rev..

[17] Wuithschick M., Birnbaum A., Witte S., Sztucki M., Vainio U., Pinna N., Rademann K., Emmerling F., Kraehnert R., Polte J. (2015). Turkevich in New Robes:
Key Questions
Answered for the Most Common Gold Nanoparticle Synthesis. ACS Nano.

[18] Daruich
De Souza C., Ribeiro Nogueira B., Rostelato M. E. C. M. (2019). Review
of the methodologies used in the synthesis gold nanoparticles by chemical
reduction. J. Alloys Compd..

[19] Thanh N. T. K., Maclean N., Mahiddine S. (2014). Mechanisms
of Nucleation and Growth
of Nanoparticles in Solution. Chem. Rev..

[20] Quinson J., Jensen K. M. Ø. (2020). From platinum
atoms in molecules to colloidal nanoparticles:
A review on reduction, nucleation and growth mechanisms. Adv. Colloid Interface Sci..

[21] Handwerk D. R., Shipman P. D., Whitehead C. B., Ozkar S., Finke R. G. (2019). Mechanism-Enabled
Population Balance Modeling of Particle Formation en Route to Particle
Average Size and Size Distribution Understanding and Control. J. Am. Chem. Soc..

[22] Mathiesen J. K., Quinson J., Blaseio S., Kjær E. T. S., Dworzak A., Cooper S. R., Pedersen J. K., Wang B., Bizzotto F., Schröder J., Kinnibrugh T. L., Simonsen S. B., Theil Kuhn L., Kirkensgaard J. J. K., Rossmeisl J., Oezaslan M., Arenz M., Jensen K. M. Ø. (2023). Chemical insights on the formation of
colloidal iridium
nanoparticles from in situ X-ray total scattering: Influence of precursors
and cations on the reaction pathway. J. Am.
Chem. Soc..

[23] Prat D., Wells A., Hayler J., Sneddon H., McElroy C. R., Abou-Shehada S., Dunn P. J. (2016). CHEM21 selection guide of classical-
and less classical-solvents. Green Chem..

[24] Rodrigues T. S., Zhao M., Yang T., Gilroy K. D., da Silva A. G. M., Camargo P. H. C., Xia Y. (2018). Synthesis of Colloidal
Metal Nanocrystals: A Comprehensive Review on the Reductants. Chem.—Eur. J..

[25] Borowskaja D. (1934). Zur Methodik
der Goldsolbereitung. Ztschr. f. Immunitatsforsch.
u. Exp. Therap..

[26] Dykman L. A., Khlebtsov N. G. (2019). Methods for chemical synthesis of colloidal gold. Russ. Chem. Rev..

[27] Turkevich J., Stevenson P. C., Hillier J. (1951). A study of the nucleation and growth
processes in the synthesis of colloidal gold. Discuss. Faraday Soc..

[28] Frens G. (1973). Controlled
nucleation for regulation of particle-size in monodisperse gold suspensions. Nature-Physical Science.

[29] Kimling J., Maier M., Okenve B., Kotaidis V., Ballot H., Plech A. (2006). Turkevich method for
gold nanoparticle synthesis revisited. J. Phys.
Chem. B.

[30] Ojea-Jimenez I., Bastus N. G., Puntes V. (2011). Influence of the Sequence of the
Reagents Addition in the Citrate-Mediated Synthesis of Gold Nanoparticles. J. Phys. Chem. C.

[31] Sivaraman S. K., Kumar S., Santhanam V. (2011). Monodisperse
sub-10 nm gold nanoparticles
by reversing the order of addition in Turkevich method - The role
of chloroauric acid. J. Colloid Interface Sci..

[32] Huang H., Toit H. d., Besenhard M. O., Ben-Jaber S., Dobson P., Parkin I., Gavriilidis A. (2018). Continuous
flow synthesis of ultrasmall gold nanoparticles in a microreactor
using trisodium citrate and their SERS performance. Chem. Eng. Sci..

[33] Iravani S., Varma R. (2019). Biofactories: engineered
nanoparticles via genetically engineered
organisms. Green Chem..

[34] Polshettiwar V., Baruwati B., Varma R. (2009). Magnetic nanoparticle-supported
glutathione:
a conceptually sustainable organocatalyst. Chem.
Commun..

[35] Sugiarto S., Aloka Weerasinghe U., Kinyanjui Muiruri J., Yu Qing Chai A., Chee Chuan Yeo J., Wang G., Zhu Q., Jun Loh X., Li Z., Kai D. (2024). Nanomaterial synthesis in deep eutectic solvents. Chem. Eng. J..

[36] Liu H., Wang S., Yang J., Zhuo R., Zhao J., Liu L., Li Y. (2024). The application
of supercritical fluid technology in
the synthesis of metal and metal oxide nanoparticles. CrystEngComm.

[37] Røjkjær
Rasmussen D., Lock N., Quinson J. (2025). Lights on the synthesis
of surfactant-free colloidal gold nanoparticles in alkaline mixtures
of alcohols and water. ChemSusChem.

[38] Quinson J. (2023). On the Importance
of Fresh Stock Solutions for Surfactant-Free Colloidal Syntheses of
Gold Nanoparticles in Alkaline Alcohol and Water Mixtures. Inorganics.

[39] Teunis T., Nota S., Schwab J. (2015). Do Corresponding Authors
Take Responsibility
for Their Work? A Covert Survey. Clin. Orthop.
Relat. Res..

[40] Marrs J., Ghomian T., Domulevicz L., McCold C., Hihath J. (2021). Gold Nanoparticle
Synthesis. J. Visualized Exp..

[41] Bolaños F., Salatino A., Osborne F., Motta E. (2024). Artificial intelligence
for literature reviews: opportunities and challenges. Artif. Intell. Rev..

[42] Roy A., Healey C., Larm N., Ishtaweera P., Roca M., Baker G. (2024). The Huge Role of Tiny Impurities
in Nanoscale Synthesis. ACS Nanoscience Au.

[43] Liz-Marzan L. M., Kagan C. R., Millstone J. E. (2020). Reproducibility in Nanocrystal Synthesis?
Watch Out for Impurities!. ACS Nano.

[44] Amri N. E., Roger K. (2020). Polyvinylpyrrolidone (PVP) impurities drastically impact the outcome
of nanoparticle syntheses. J. Colloid Interface
Sci..

[45] Fievet F., Ammar-Merah S., Brayner R., Chau F., Giraud M., Mammeri F., Peron J., Piquemal J. Y., Sicard L., Viau G. (2018). The polyol
process: a unique method for easy access to metal nanoparticles
with tailored sizes, shapes and compositions. Chem. Soc. Rev..

[46] Quinson J., Bucher J., Simonsen S. B., Kuhn L. T., Kunz S., Arenz M. (2019). Monovalent alkali cations:
simple and eco-friendly stabilizers for
surfactant-free precious metal nanoparticle colloids. ACS Sustainable Chem. Eng..

[47] Quinson J., Aalling-Frederiksen O., Dacayan W. L., Bjerregaard J. D., Jensen K. D., Jørgensen M. R.
V., Kantor I., Sørensen D. R., Theil Kuhn L., Johnson M. S., Escudero-Escribano M., Simonsen S. B., Jensen K. M. Ø. (2023). Surfactant-free colloidal
syntheses of gold-based nanomaterials in alkaline water and mono-alcohol
mixtures. Chem. Mater..

[48] Andersen K. J., Varga M., Smolska A., Nordhal G., Jensen J. H., Moreno R., Bøjesen E. D., Anker A. S., Quinson J. (2025). Positive thinking:
counter-cations effects in colloidal syntheses of gold nanoparticles. Nano Lett..

[49] Strmcnik D., Kodama K., van der Vliet D., Greeley J., Stamenkovic V. R., Markovic N. M. (2009). The role of non-covalent
interactions in electrocatalytic
fuel-cell reactions on platinum. Nat. Chem..

[50] Jimenez-Ruiz A., Perez-Tejeda P., Grueso E., Castillo P. M., Prado-Gotor R. (2015). Nonfunctionalized
Gold Nanoparticles: Synthetic Routes and Synthesis Condition Dependence. Chem.—Eur. J..

[51] Quinson J., Kacenauskaite L., Bucher J., Simonsen S. B., Theil
Kuhn L., Oezaslan M., Kunz S., Arenz M. (2019). Controlled Synthesis
of Surfactant-Free Water-Dispersible Colloidal Platinum Nanoparticles
by the Co4Cat Process. ChemSusChem.

[52] Parveen R., Ullah S., Sgarbi R., Tremiliosi-Filho G. (2019). One-pot ligand-free
synthesis of gold nanoparticles: The role of glycerol as reducing-cum-stabilizing
agent. Colloids Surf., A.

[53] Deraedt C., Salmon L., Gatard S., Ciganda R., Hernandez R., Ruiz J., Astruc D. (2014). Sodium borohydride
stabilizes very
active gold nanoparticle catalysts. Chem. Commun..

[54] Jæger F., Pedersen A. A., Wacherhausen P. S., Smolska A., Quinson J. (2025). Surfactant-free
gold nanoparticles synthesized in alkaline water-ethanol mixtures:
leveraging lower grade chemicals for size control of active nanocatalysts. RSC Sustainability.

[55] Reichenberger S., Marzun G., Muhler M., Barcikowski S. (2019). Perspective
of Surfactant-free Colloidal Nanoparticles in Heterogeneous Catalysis. ChemCatChem..

[56] Kim K. K., Kwon H. J., Shin S. K., Song J. K., Park S. M. (2013). Stability
of uncapped gold nanoparticles produced by laser ablation in deionized
water: The effect of post-irradiation. Chem.
Phys. Lett..

[57] Balachandran A., Sreenilayam S. P., Madanan K., Thomas S., Brabazon D. (2022). Nanoparticle
production via laser ablation synthesis in solution method and printed
electronic application—A brief review. Results Eng..

[58] Fokam H. K., Smolska A., Quinson J. (2025). Surfactant-free NaBH_4_-mediated
synthesis of gold nanoparticles in water at room temperature: fine
size control for active nanocatalysts. ChemRxiv.

[59] Rambaran T., Schirhagl R. (2022). Nanotechnology
from lab to industry - a look at current
trends. Nanoscale Advances.

[60] Sánchez
Jiménez A., Puelles R., Perez-Fernandez M., Barruetabeña L., Jacobsen N. R., Suarez-Merino B., Micheletti C., Manier N., Salieri B., Hischier R., Tsekovska R., Handzhiyski Y., Bouillard J., Oudart Y., Galea K. S., Kelly S., Shandilya N., Goede H., Gomez-Cordon J., Jensen K. A., van Tongeren M., Apostolova M. D., Llopis I. R. (2022). Safe­(r) by design guidelines
for the nanotechnology industry. NANO.

[61] Oestreicher V., Garcia C. S., Soler-Illia G., Angelome P. C. (2019). Gold Recycling at
Laboratory Scale: From Nanowaste to Nanospheres. ChemSusChem.

[62] Quinson J., Mathiesen J. K., Schroder J., Dworzak A., Bizzotto F., Zana A., Simonsen S. B., Kuhn L. T., Oezaslan M., Jensen K. M. O. (2020). Teaching old precursors new tricks: Fast room
temperature synthesis of surfactant-free colloidal platinum nanoparticles. J. Colloid Interface Sci..

[63] Fokam, H. K. ; Smolska, A. ; Quinson, J. NaBH_4_-mediated syntheses of colloidal gold nanocatalysts in water: are additives really needed? ChemRxiv 2025, 10.26434/chemrxiv-2025-h0hpb.

[64] Quinson J., Neumann S., Wannmacher T., Kacenauskaite L., Inaba M., Bucher J., Bizzotto F., Simonsen S. B., Theil Kuhn L., Bujak D., Zana A., Arenz M., Kunz S. (2018). Colloids for Catalysts:
A Concept for the Preparation
of Superior Catalysts of Industrial Relevance. Angew. Chem., Int. Ed..

[65] Khan T., Ullah N., Khan M. A., Mashwani Z. U. R., Nadhman A. (2019). Plant-based
gold nanoparticles; a comprehensive review of the decade-long research
on synthesis, mechanistic aspects and diverse applications. Adv. Colloid Interface Sci..

[66] Dong H., Chen Y. C., Feldmann C. (2015). Polyol synthesis
of nanoparticles:
status and options regarding metals, oxides, chalcogenides, and non-metal
elements. Green Chem..

[67] Quinson J., Simonsen S. B., Theil
Kuhn L., Arenz M. (2021). Commercial spirits
for surfactant-free syntheses of electro-active platinum nanoparticles. Sustainable Chemistry.

[68] Panagopoulos D., Alamdari A. A., Quinson J. (2025). Surfactant-free
colloidal gold nanoparticles:
room temperature synthesis, size control and opportunities for catalysis. Mater. Today Nano.

[69] Læsaa S., Smolska A., Ceccato M., Dražević E., Quinson J. (2025). Recycling of solvent
and reducing agent in the synthesis
of copper-based particles using 2-ethylanthraquinone. Sustainabiity Science Technollogy.

[70] Rossi L. M., Fiorio J. L., Garcia M. A. S., Ferraz C. P. (2018). The role and fate
of capping ligands in colloidally prepared metal nanoparticle catalysts. Dalton Trans..

[71] Johnson P., Trybala A., Starov V., Pinfield V. J. (2021). Effect of synthetic
surfactants on the environment and the potential for substitution
by biosurfactants. Adv. Colloid Interface Sci..

[72] Quinson J., Kunz S., Arenz M. (2023). Surfactant-free
colloidal syntheses
of precious metal nanoparticles for improved catalysts. ACS Catal..

[73] Van
Gordon K., Baúlde S., Mychinko M., Heyvaert W., Obelleiro-Liz M., Criado A., Bals S., Liz-Marzán L., Mosquera J. (2023). Tuning the Growth of Chiral Gold Nanoparticles Through
Rational Design of a Chiral Molecular Inducer. Nano Lett..

[74] Heuer-Jungemann A., Feliu N., Bakaimi I., Hamaly M., Alkilany A., Chakraborty I., Masood A., Casula M. F., Kostopoulou A., Oh E. (2019). The Role of Ligands
in the Chemical Synthesis and Applications
of Inorganic Nanoparticles. Chem. Rev..

[75] Losch P., Huang W. X., Goodman E. D., Wrasman C. J., Holm A., Riscoe A. R., Schwalbe J. A., Cargnello M. (2019). Colloidal
nanocrystals for heterogeneous catalysis. Nano
Today.

[76] Koczkur K. M., Mourdikoudis S., Polavarapu L., Skrabalak S. E. (2015). Polyvinylpyrrolidone
(PVP) in nanoparticle synthesis. Dalton Trans..

[77] Wang Y., Ren J. W., Deng K., Gui L. L., Tang Y. Q. (2000). Preparation
of tractable platinum, rhodium, and ruthenium nanoclusters with small
particle size in organic media. Chem. Mater..

[78] Bizzotto F., Quinson J., Schröder J., Zana A., Arenz M. (2021). Surfactant-free
colloidal strategies for highly dispersed and active supported IrO_2_ catalysts: Synthesis and performance evaluation for the oxygen
evolution reaction. J. Catal..

[79] Varga M., Quinson J. (2025). Fewer, but better:
on the benefits for surfactant-free
colloidal syntheses of nanomaterials. ChemistrySelect.

[80] Halford G., Hertle S., Nambiar H., Personick M. (2025). Using Electrochemistry
to Benchmark, Understand, and Develop Noble Metal Nanoparticle Syntheses. ACS Nanosci. Au.

[81] Panariello L., Radhakrishnan A. N. P., Papakonstantinou I., Parkin I. P., Gavriilidis A. (2020). Particle Size
Evolution during the Synthesis of Gold Nanoparticles Using In Situ
Time-Resolved UV-Vis Spectroscopy: An Experimental and Theoretical
Study Unravelling the Effect of Adsorbed Gold Precursor Species. J. Phys. Chem. C.

[82] Anker, A. S. ; Jensen, J. H. ; Gonzalez-Duque, M. ; Moreno, R. ; Smolska, A. ; Juelsholt, M. ; Hardion, V. ; Jorgensen, M. R. V. ; Faina, A. ; Quinson, J. Autonomous nanoparticle synthesis by design. arXiv 2025, 10.48550/arXiv.2505.13571. PMC12961950 41705948

[83] Mints V., Pedersen J., Bagger A., Quinson J., Anker A., Jensen K., Rossmeisl J., Arenz M. (2022). Exploring the composition
space of high-entropy alloy nanoparticles for the electrocatalytic
H_2_/CO oxidation with Bayesian optimization. ACS Catal..

[84] Jensen T. B., Saugbjerg J. R., Henriksen M. L., Quinson J. (2024). Towards the automation
of nanoparticle syntheses: The case study of gold nanoparticles obtained
at room temperature. Colloids Surf., A.

[85] Zhao H., Chen W., Huang H., Sun Z., Chen Z., Wu L., Zhang B., Lai F., Wang Z., Adam M. (2023). A robotic platform for
the synthesis of colloidal nanocrystals. Nature
Synthesis.

[86] Epps R. W., Abolhasani M. (2021). Modern nanoscience: Convergence of
AI, robotics, and
colloidal synthesis. Applied Physics Reviews.

[87] Dallinger D., Kappe C. O. (2017). Why flow means green
- Evaluating the merits of continuous
processing in the context of sustainability. Current Opinion in Green and Sustainable Chemistry.

[88] Quinson J. (2023). Room Temperature
Surfactant-Free Syntheses of Gold Nanoparticles in Alkaline Mixtures
of Water and Alcohols: A Model System to Introduce Nanotechnology
and Green Chemistry to Future Chemists and Engineers. J. Chem. Educ..

[89] Horton M., Woods-Robinson R. (2021). Addressing
the critical need for open experimental
databases in materials science. Patterns.

